# A Lead *ANRIL* Polymorphism Is Associated with Elevated CRP Levels in Periodontitis: A Pilot Case-Control Study

**DOI:** 10.1371/journal.pone.0137335

**Published:** 2015-09-08

**Authors:** Wijnand J. Teeuw, Marja L. Laine, Sergio Bizzarro, Bruno G. Loos

**Affiliations:** Department of Periodontology, Academic Centre for Dentistry Amsterdam (ACTA), University of Amsterdam and VU University Amsterdam, Amsterdam, the Netherlands; UNC School of Dentistry, University of North Carolina-Chapel Hill, UNITED STATES

## Abstract

Elevated high sensitive C-reactive protein (hsCRP) is a marker for systemic inflammation and a risk marker for atherosclerotic cardiovascular disease (ACVD), and has also been associated with periodontitis. Inter-individual variation for hsCRP in periodontitis has been shown. *ANRIL* is the strongest genetic susceptibility locus for both periodontitis and ACVD, and it is speculated that genetic variation in *ANRIL* may modulate inflammatory processes. Therefore, we explored the possible association between hsCRP plasma levels and a leading *ANRIL* single nucleotide polymorphism (SNP) in periodontitis patients and controls. 171 healthy subjects with North European descent (115 periodontitis and 56 controls) were included in this case-control study. hsCRP levels were determined and subjects were genotyped for the leading *ANRIL* SNP rs1333048. In a multivariate analysis, periodontitis, female gender, increasing BMI and homozygosity for the major allele (AA-genotype) of rs1333048 were significantly associated with elevated hsCRP plasma levels (p = 0.012, p = 0.004, p = 0.007 and p = 0.003, respectively). Periodontitis patients with rs1333048 AA-genotype showed higher levels of hsCRP than those carrying the minor C allele (median: 4.5 mg/L vs. 1.6 mg/L, p_adjusted_ = 0.007). This study is the first to show that, in addition to gender and BMI, also a leading SNP in *ANRIL* is explanatory for inter-individual variation in hsCRP levels in periodontitis patients of North European descent.

## Introduction

Periodontitis is a complex, chronic inflammatory disease, resulting in loss of connective tissue and alveolar bone support of the teeth [1]. It is the major cause of tooth loss in adults above 40 years and affects human populations worldwide at prevalence rates up to 10–20% for the most severe forms [2,3]. Periodontitis is associated with moderately increased concentration of high sensitive C-reactive protein (hsCRP) in the blood circulation [4]. These elevated levels of hsCRP in periodontitis are ‘dose dependent’ with higher levels in severe forms of periodontitis, compared to moderate forms and periodontal health and range between 1–4 mg/L [5]. Periodontal therapy reduces periodontal inflammation and recent meta-analyses are highly suggestive that this reduction results in lower plasma levels of hsCRP (overall: -0.5 mg/L) [6].

Although on average periodontitis patients show elevated hsCRP, variations in levels have been shown both between patients and between populations [4,6]. Also the reduction of hsCRP levels after periodontal treatment varies substantially between study groups [6], suggesting that additionally several other factors may influence the systemic inflammatory response in patients suffering from periodontitis. Interestingly, hsCRP is also widely accepted as key marker of atherosclerosis and is strongly associated with increased risk for atherosclerotic cardiovascular disease (ACVD); levels of 1–3 mg/L and >3 mg/L are considered to give medium and high risk for ACVD, respectively [7,8]. In this context, also increasing BMI and female gender have been suggested as factors associated with chronically elevated hsCRP [9,10].

In general, inflammatory responses are modulated by specific polymorphisms in inflammatory genes [11]. We speculate that polymorphisms in the *ANRIL* locus on chromosome 9p21 may be involved. *ANRIL* is a genetic risk factor for several conditions with inflammatory components in Caucasians, and is the strongest genetic susceptibility locus for periodontitis [12,13] and several types of ACVD, like coronary artery disease (CAD), myocardial infarction, abdominal aortic aneurysm and intracranial aneurysm [14–16]. It has been shown that the disease-associated single nucleotide polymorphisms (SNPs) of chromosome 9p21 have been associated with the expression of *ANRIL* [17]. In particular, the CAD-associated polymorphisms within the core risk haplotype region have been shown to regulate *ANRIL* expression *in vitro* [18] and also *in vivo* [19]. But so far, the function of *ANRIL* in relation to periodontitis and ACVD is not fully understood. At least one pathway is known, *ANRIL* is regulated by STAT1 signaling [18], a pathway that mediates response to inflammation upon stimulation of the pro-inflammatory cytokine interferon-γ. Furthermore, *ANRIL* transcription was up-regulated in gingival tissues by bacterial infection [13]. *ANRIL* may therefore be an important regulator of inflammatory and immune reactions, such as in the pathophysiology of periodontitis, ACVD, diabetes mellitus and cancer [19]. If so, variation in plasma hsCRP (as endpoint and reporter molecule of a variety of inflammatory pathways) may reflect inter-individual heterogeneity of *ANRIL* activity based on genetic variation. Therefore, we hypothesize that the observed variation in hsCRP levels in periodontitis patients might be associated with genetic variation in the *ANRIL* gene in addition to other factors and may explain inter-individual differences in hsCRP levels among periodontitis patients.

The aim of the present explorative pilot case-control study was to investigate the possible association between levels of hsCRP and the leading ACVD- and periodontitis-associated gene polymorphism (*ANRIL* rs1333048, [12,13]) in Caucasian periodontitis patients and controls.

## Materials and Methods

### Study population

**Fig 1:**
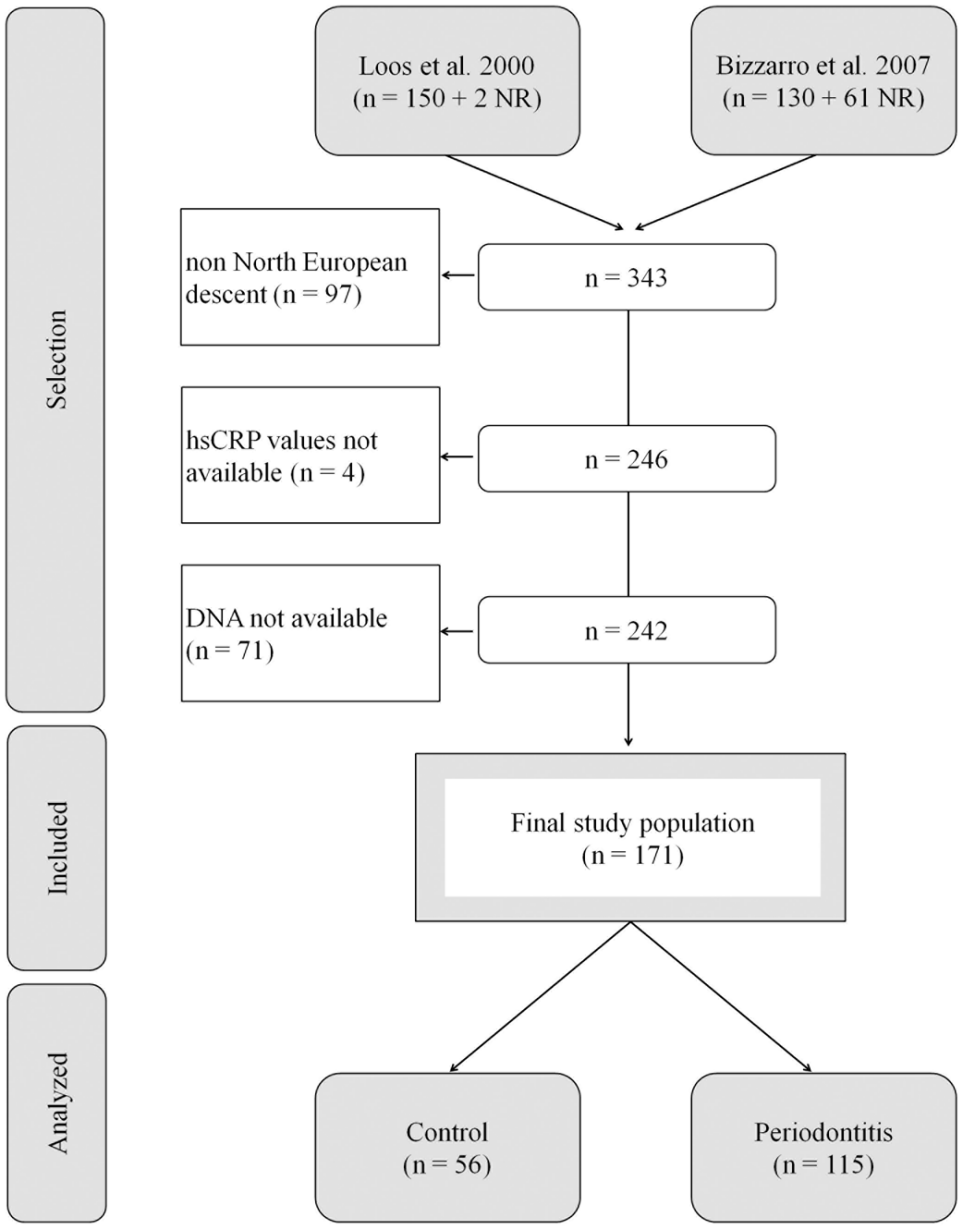
Flowchart of patient selection. Of 343 subjects, 171 were suitable for analysis. NR: additional patients obtained during the study period. However, due to missing data, not reported in that particular study.

A total of 171 subjects from North European descent, including 115 patients with periodontitis and 56 controls, were suitable to participate in this pilot study. These individuals were retrieved from a total of 343 individuals from two previous studies [20,21] (Fig 1). For all study subjects, age, gender, body mass index (BMI) and current smoking were extracted out of the databases [20,21]. Briefly, referred periodontitis patients were recruited during their first visit at the Department of Periodontology of the Academic Centre Dentistry Amsterdam (ACTA). Clinical measurements were performed at six sites per tooth and a full radiographic status was available to analyze interproximal alveolar bone levels. Patients were classified as suffering from periodontitis using the CDC-AAP case definition for moderate to severe periodontitis [22]; patients showed at least 2 interproximal sites with attachment loss (AL) ≥4 mm on different teeth in conjunction with alveolar bone loss on the basis of peri-apical radiographs. All patients were untreated and showed generalized bleeding on probing. Controls were selected among subjects registered for restorative dental procedures or who visited ACTA for regular dental check-ups. Control subjects were included if they were not missing more than one tooth per quadrant (3rd molar excluded) and no probing pocket depth (PPD) ≥3 mm. These subjects showed on dental bitewing radiographs ≤1-year-old a distance between the cemento-enamel junction and the alveolar bone crest of ≤3 mm on all teeth.

Exclusion criteria for the current study subjects were: (i) the presence of a systemic disease, (ii) recent history of any acute or chronic infection, periodontitis not included, (iii) systemic antibiotic treatment within the last 3 months, (iv) the use of any medication (including sporadic NSAID’s), (v) pregnancy or lactation, vi) non North European descent, and vii) no DNA available (Fig 1).

The Medical Ethical Committee of the Academic Medical Center, Amsterdam, approved this study and all participants gave their written informed consent to participate.

### Analysis of hsCRP plasma levels

Non-fasting, venous blood was collected from each subject in EDTA containing tubes between 8:30 and 11:30am. Plasma levels of hsCRP were determined using a high sensitivity nephelometric method on the BN ProSpec analyzer (Dade Behring, Marburg, Germany). The detection limit was 0.3 mg/L, linearity was from 0.3 to 230 mg/L, and the coefficient of variation was <3% at a concentration of 2 mg/L. Subjects with hsCRP values <0.3 mg/L were regarded sero-negative [20,21].

### Analysis of gene polymorphisms

For all participants, the lead and validated periodontitis- and ACVD-associated intronic SNP rs1333048 in the core risk region of the *ANRIL* gene [12,13,23] was investigated. This SNP is also in strong linkage disequilibrium with other representative SNPs that are associated with both periodontitis and ACVD [24]. The polymorphism was assayed at the Institute for Clinical Molecular Biology of the Christian Albrechts University, Kiel, Germany, with TaqMan (Applied Biosystems [ABI], Foster City, CA, USA) on an automated platform. In brief, genomic DNA was arrayed on 96-well or 384-well plates. Samples were amplified with ABI 9700 PCR machines and fluorescence was measured with ABI 7700 fluorometer. The assay included two locus-specific PCR primers that flank the SNP of interest, and two allele-specific oligonucleotide probes (TTCATGCTATTTTGAGGAGATGTTT[A/C]AATGTCGAATTATTGAAATATTTAT (Context Sequence [VIC/FAM]). For the polymorphism assayed, a PCR-protocol was used as follows: a) activation: 95°C, 10 min, 1 cycle; b) denaturation (95°C, 15s), annealing, elongation, nucleolytic cleavage of hybridized probes (60°C, 1 min), 45 cycles; c) storage: 4°C. For the endpoint measurement the ABIPrism 7900 HT Sequence Detection System was used. The assay had a call rate of 100%. The A allele of rs1333048 has been determined as the major allele, while the C allele is the minor allele [13,15]; subjects were genotyped as AA, AC or CC.

### Data analyses

Data analyses were performed with the SPSS 18.0 package (SPSS Inc., Chicago, IL, USA). Means, standard deviations, medians, interquartile ranges (IQR) and frequency distributions were calculated. Because of the non-normal distribution, hsCRP plasma levels were log transformed for all calculations. The general characteristics of the study population, hsCRP levels, genotype and allelic distribution within the study population were compared with parametric and non-parametric tests (ANOVA and chi-squared test). In addition, boxplots were generated. Where applicable, analyses were corrected for multiple testing (Bonferroni). Deviations from Hardy-Weinberg equilibrium were tested in the control group by using a chi-squared test and a type I error level of 0.05. Odds Ratios (OR) and confidence intervals (CI), adjusted for age, gender, smoking habits and BMI, were calculated. Logistic regression analysis was performed. Significance was assessed by a Wald test and by a likelihood-ratio test. Univariate analysis was performed (ANCOVA), using hsCRP levels as the outcome parameter and periodontitis as a fixed factor. Age, gender, smoking habits, BMI, and carriage of the minor allele (AC or CC) of rs1333048 were included as covariates. For all analyses, the significance level was set to p<0.05.

## Results

Demographic, clinical and genotypic characteristics of the study population are presented in Table 1. The mean ages for the control and periodontitis group were 43.9, 45.5 years, respectively. Among periodontitis patients, 53.9% were smokers, while this was 30% among controls (*p* = 0.004). The gender distribution was not significantly different among the control and periodontitis groups (males: 46.4% and 35.7%, respectively). Periodontitis patients had significantly less teeth than controls (*p*<0.001), and the periodontitis group presented on average 6.7 teeth with ≥50% alveolar bone loss. Controls had no alveolar bone loss.

**Table 1:** General characteristics of the study population.

	Control (n = 56)	Periodontitis (n = 115)	*P*-value
**Background characteristics**			
Age	43.9 ± 13.2	45.5 ± 9.9	0.430
Gender (males)	26 (46.4%)	41 (35.7%)	0.175
BMI (kg/m^2^)	24.9 ± 3.5	24.5 ± 3.6	0.567
# teeth present	27.7 ± 1.9	25.8 ± 3.2	<0.001
# teeth with ≥50% alveolar bone loss	-	6.7 ± 5.7	NA
# patients with ≥7 teeth with ≥50% alveolar bone loss	-	51 (44.3%)	NA
# Smokers	17 (30.4%)	62 (53.9%)	0.004
**Systemic inflammatory marker**			
hsC-reactive protein (mg/L), median (IQR)	1.3 (0.6–2.5)	1.8 (0.8–4.5)	0.031
***ANRIL* rs1333048 (A>C)**			
Genotypes frequency			
A/A	20 (35.7%)	23 (20.0%)	
A/C	26 (46.4%)	60 (52.2%)	0.065
C/C	10 (17.9%)	32 (27.8%)	
MAF (C-allele)	0.41	0.54^a^	0.026
C allele carrier (%)	36 (64.3%)	92 (80.0%)	0.026

^a^ Carriage of the C-allele was significantly associated with periodontitis: adjusted OR = 2.56 (95%CI: 1.20–5.50), *p* = 0.015

MAF = Minor Allele Frequency; NA = Not applicable

hsCRP was significantly different among the two groups (*p* = 0.031, Table 1). The median levels of hsCRP levels for controls and periodontitis were 1.3 mg/L and 1.8 mg/L, respectively.

Genotyping results showed no significant deviation from the Hardy-Weinberg equilibrium in the control group. There was no significant difference in the genotype frequencies of *ANRIL* rs1333048 between periodontitis and controls (*p* = 0.065, Table 1). However, minor allele frequencies and carriage of the C-allele of rs1333048 (genotypes AC and CC combined) was significantly associated with periodontitis (adjusted OR = 2.56 [95% CI: 1.20–5.50], *p* = 0.015).

### Univariate analysis of hsCRP levels

Univariate analysis was performed to explore the relation of several covariates with hsCRP levels (Table 2). Including the potential confounders, periodontitis was significantly associated with elevated hsCRP plasma levels (*p* = 0.012). After adjustment for the included covariates, the adjusted mean hsCRP levels for the control and periodontitis group were 1.19 mg/L (CI: 0.90–1.57), and 1.86 mg/L (CI: 1.11–2.22), *p* = 0.012, respectively. In addition, the covariates gender, BMI and genotype were significant: females (*p* = 0.004), individuals with increased BMI (*p* = 0.007) and subjects homozygous for the major allele (AA-genotype) of rs1333048 showed increased plasma concentrations of hsCRP (*p* = 0.003).

**Table 2:** Univariate analysis for hsCRP levels.

	*F*-ratio	*p*-value
Intercept	6.83	0.010
Periodontitis ^a^	6.50	0.012
Age	2.82	0.095
Gender (female)	8.63	0.004
Smoking	3.03	0.084
BMI	7.58	0.007
*ANRIL* rs1333048 (AA-genotype)	9.00	0.003

^a^ hsCRP means and 95% Confidence Interval (CI) adjusted for all covariates: Control: 1.19 mg/L, CI: 0.90–1.57; Periodontitis: 1.86 mg/L, CI: 1.11–2.22)

**Fig 2:**
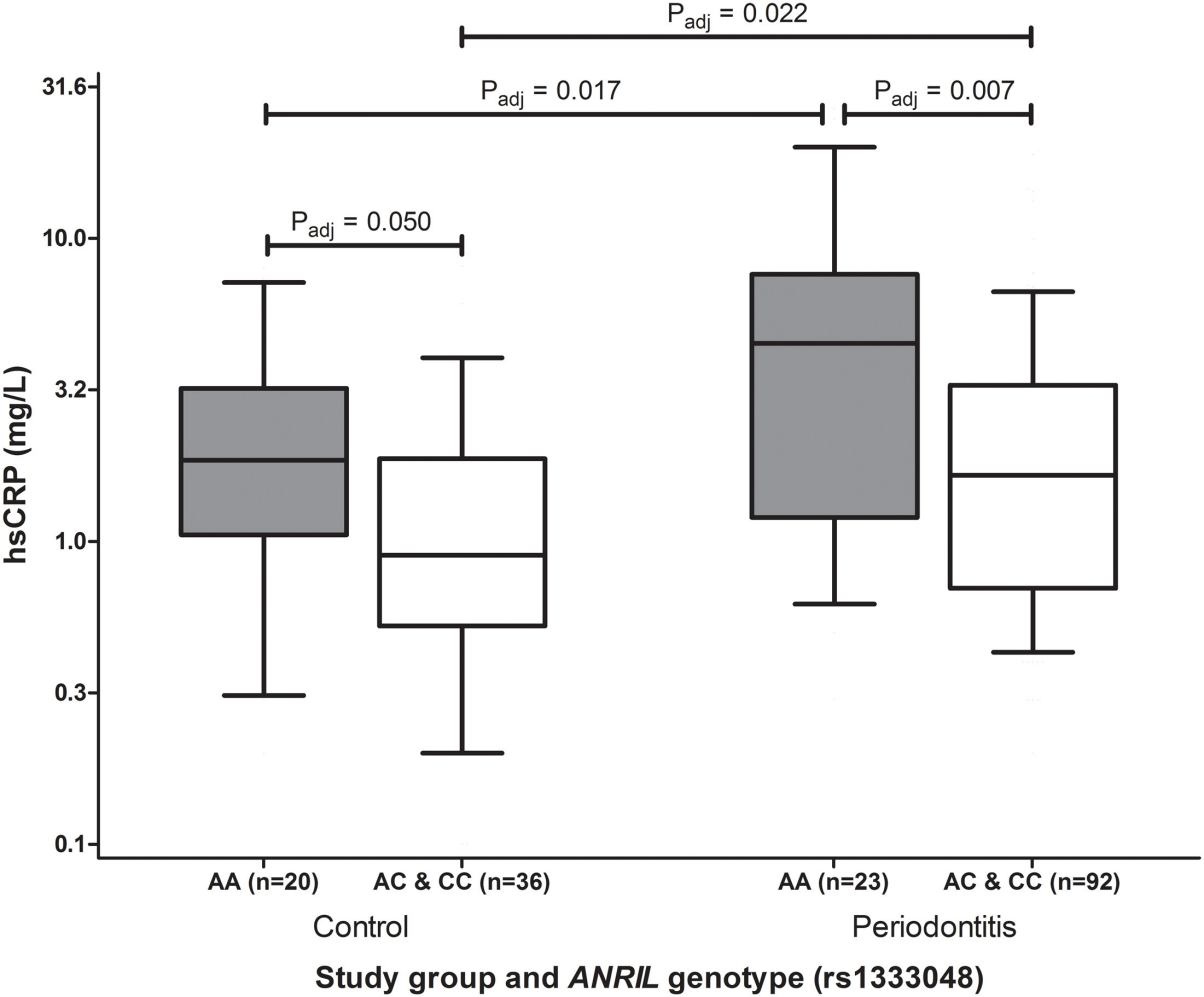
Boxplots of hsCRP plasma levels in subjects with or without periodontitis according to their genotype of *ANRIL* (rs1333048). Homozygosity for the major allele (AA-genotype) of *ANRIL* (rs1333048) is associated with significantly elevated hsCRP plasma levels in patients with periodontitis. Boxes represent interquartile ranges (IQR) and median values are indicated by the horizontal line within the box. Whiskers represent the 10^th^ and 90^th^ percentiles. P-values are adjusted for age, gender, smoking and BMI. Y-axis is in logarithmic scale.

The association of the *ANRIL* genotype with hsCRP levels is a new finding. Therefore, we explored the hsCRP levels in relation to periodontitis, subgrouped by carriership of the minor allele. Fig 2 shows that subjects with periodontitis and homozygous for the major allele (AA-genotype) of rs1333048 have significantly elevated hsCRP levels (4.5 mg/L [median]; IQR: 1.2–7.6 mg/L) compared to subjects with periodontitis and carriage of the minor allele (AC and CC genotypes) (1.6 mg/L [median; IQR: 0.7–3.3 mg/L; *p* = 0.007] and healthy controls homozygous for the major allele (1.9 mg/L [median]; IQR: 1.1–3.0 mg/L; *p* = 0.017). A trend for intragroup differences in hsCRP levels between subjects of the control group homozygous for the major allele and subjects carrying the minor allele was also observed (*p* = 0.050). Notably, carriage of the minor allele of rs1333048 is associated with relative low hsCRP levels, nevertheless the periodontitis patients with genotypes AC or CC showed higher hsCRP levels than the corresponding individuals in the control group (periodontitis: 1.6 mg/L [median]; IQR: 0.7–3.2 mg/L; control: 0.9 mg/L [median]; IQR: 0.6–1.7 mg/L; *p* = 0.022).

## Discussion

The present pilot study aimed to investigate the possible association between hsCRP plasma levels and potential confounding factors, including the leading polymorphism in *ANRIL*, in periodontitis patients and controls. *ANRIL* is an important genetic locus as it is associated with several chronic systemic conditions, including ACVD and periodontitis. The main finding of this study is that not only periodontitis, BMI and female gender are related with elevated CRP: also the AA-genotype of *ANRIL* (rs1333048) is associated with significantly elevated hsCRP plasma levels in patients with periodontitis. While periodontitis, female gender and elevated BMI have been associated with increased hsCRP in the literature [4,9,10], this is the first study that shows that a SNP in *ANRIL* is also associated with hsCRP levels in periodontitis patients. Interestingly, a trend was also seen for controls, but this did not reach statistical significance (*p* = 0.050). The observation suggests that among other and currently unknown factors, the *ANRIL* locus may be involved in ‘generic’ inflammatory processes of immune-mediated diseases with hsCRP as final biomarker at one or several pathways. The challenge is to turn the increasing knowledge of pleiotropic pathways to clinical relevance [25].

We recognize that periodontitis is a complex disease with multiple causal factors playing a role simultaneously [26]. These can be grouped in genetic, bacteria and lifestyle related factors, including diet, systemic diseases, notably diabetes, and other factors as of yet unknown. Currently, more genetic loci, in addition to *ANRIL*, shared between ACVD and periodontitis have been identified: *PLASMINOGEN* and a conserved noncoding element within *CAMTA1* upstream of *VAMP3* [27,28]. Recent experimental and epidemiological studies suggested that *ANRIL*, *VAMP3* and *PLASMINOGEN* are involved in several regulatory networks that relate to glucose and fatty acid metabolism, host-microbiome interactions and TGF-β signaling, providing evidence for a mechanistic link between ACVD, periodontitis, obesity and inflammation [27–30]. Any disturbances in these pathways by genetic variants may be a common pathogenic trait of ACVD and periodontitis.

Previous studies showed that carriership of the minor allele of rs1333048 is associated with ACVD and periodontitis. In the present study we corroborate this; the C-allele was more frequent in periodontitis patients than controls. However, the clinical effects of this confirmed association have never been reported, although functionality of the SNP is suggested *in vitro*/*ex vivo* experiments [27]. We show here that, in the inflammatory disease periodontitis, C-carriage is associated with lower plasma levels of hsCRP compared with those that had the AA-genotype. The role of CRP in periodontitis is not yet established. CRP is regarded as a sensitive, non-specific marker of inflammation. However, although chronic elevated hsCRP levels are considered as a prognostic risk marker for ACVD [7,8], in acute infection or inflammation the role of CRP might be protective [31]. In that sense, genetic variations causing low CRP levels might play an etiologic role. It is known that CRP can inhibit the alternative complement pathway by binding factor H [32–35]. This results in restricted complement activation favoring opsonization and not in the formation of the membrane attack complex, subsequently leading to a less strong inflammatory response and therefore less inflammation derived tissue damage [31]. Nevertheless, we have to realize that for certain cases, even with high levels of CRP, other factors might play a more dominant role in the onset and/or progression of periodontal disease.

For example smoking is known to be a strong etiologic factor for periodontitis. Interestingly, it was in the current study not related to elevated hsCRP plasma levels in periodontitis patients and controls. A possible explanation could be that the effect of smoking depends on the number of cigarettes consumed per day (heavy or light smoking) [36]. Furthermore, the effect of smoking on systemic inflammation is not straight forward and the results of several studies are contradictory [37,38]. It is suggested, that cigarette smoking may affect inflammation via other pathways than elevated hsCRP levels; an association between smoking status and the presence of advanced atherosclerotic lesions but not with hsCRP plasma levels has been shown [39].

In this pilot study we show for the first time that, in addition to gender and BMI, a leading SNP in *ANRIL* is associated with variations in hsCRP plasma levels in periodontitis patients from North European descent. Replication studies in other Caucasian and non-Caucasian periodontitis patients are needed as well as biochemical studies are necessary to explain the role of *ANRIL* as pleiotrophic gene affecting inflammatory pathways of different immune-mediated diseases [25], like periodontitis and ACVD.

## Supporting Information

S1 Table STROBE Checklist for Case-Control study. (DOC)
